# *MsNRAMP2* Enhances Tolerance to Iron Excess Stress in *Nicotiana tabacum* and MsMYB Binds to Its Promoter

**DOI:** 10.3390/ijms241411278

**Published:** 2023-07-10

**Authors:** Run-Tian Li, Yun-Jiao Yang, Wen-Jun Liu, Wen-Wei Liang, Miao Zhang, Shi-Chen Dong, Yong-Jun Shu, Dong-Lin Guo, Chang-Hong Guo, Ying-Dong Bi

**Affiliations:** 1 College of Life Science and Technology, Harbin Normal University, Harbin 150025, China; lrt_9703@163.com (R.-T.L.); yunjiao0606@163.com (Y.-J.Y.); lwj8767@163.com (W.-J.L.); liangwenwei5@163.com (W.-W.L.); zhaomiaokid669@163.com (M.Z.); dsc19980519@163.com (S.-C.D.); syjun2003@126.com (Y.-J.S.); kaku_2008@163.com (C.-H.G.); 2 Institute of Crops Tillage and Cultivation, Heilongjiang Academy of Agricultural Sciences, Harbin 150086, China

**Keywords:** *MsNRAMP2*, iron excess, *MsMYB*, alfalfa, promoter, tobacco

## Abstract

Iron(Fe) is a trace metal element necessary for plant growth, but excess iron is harmful to plants. Natural resistance-associated macrophage proteins (NRAMPs) are important for divalent metal transport in plants. In this study, we isolated the *MsNRAMP2* (MN_547960) gene from alfalfa, the perennial legume forage. The expression of *MsNRAMP2* is specifically induced by iron excess. Overexpression of *MsNRAMP2* conferred transgenic tobacco tolerance to iron excess, while it conferred yeast sensitivity to excess iron. Together with the *MsNRAMP2* gene, *MsMYB* (MN_547959) expression is induced by excess iron. Y1H indicated that the MsMYB protein could bind to the “CTGTTG” cis element of the *MsNRAMP2* promoter. The results indicated that *MsNRAMP2* has a function in iron transport and its expression might be regulated by *MsMYB*. The excess iron tolerance ability enhancement of *MsNRAMP2* may be involved in iron transport, sequestration, or redistribution.

## 1. Introduction

Alfalfa (*Medicago sativa* L.), a perennial herb belonging to the legume family, has been used as a worldwide forage crop because it is rich in nutrients, including protein, dietary fiber, vitamins, and minerals [1]. Alfalfa also has potential economic value in the phytoremediation of soil contamination. Soil contamination with toxic metal elements is an increasing environmental problem, posing serious threats to living organisms. Alfalfa has a large biomass productivity, high tolerance, and strong uptake capacity to potentially toxic elements [2]. In the health risk assessment through determining the bioaccumulation of iron (Fe) in forages grown in soil irrigated with city effluent, the maximum value of daily intake of iron was observed in *Medicago sativa* [3]. Although alfalfa has been widely adopted in phytoremediation technology for the remediation of contaminated soils, there are relatively few studies investigating the heavy metals alleviation possibilities and the mechanisms of heavy metal uptake and excess metal tolerance in alfalfa [4].

Various micronutrients (including iron, copper, zinc, etc.) are essential in plant growth and development and are additionally essential for the basic metabolic processes of forage crops [5]. As an essential trace element for plants, Fe is a cofactor of more than 300 enzymes and plays a role in important metabolic processes such as photosynthesis, respiration, and amino acid biosynthesis [6]. Iron has a crucial role in photosynthetic organisms which is a required cofactor for the operation of photosynthetic functions [7]. However, iron excess significantly inhibits plant growth and development and results in serious metabolic disorders that affect photosynthesis, respiration, and general plant fitness, which have direct consequences on crop production and indirectly affect human health [8]. Excessive iron in plants catalyzes the generation of radicals by the Fenton reaction (Fe^2+^ + H_2_O_2_ → Fe^3+^ + OH^−^ + OH), which attacks and damages cellular macromolecules causing serious oxidative damage [9]. At the same time, the sensitivity of photosystem II to photoinhibition is enhanced, resulting in a decrease in the photosynthetic rate [10].

Iron is not evenly distributed in soil due to geological and human activities; low content is 1–5 mg/kg and high content can be up to 1000 mg/kg [11]. Anthropogenic input, mainly mining activities, has increased the Fe content in the environment which can influence the metal contents in plants and ultimately delivery into the food chain, thereby risking the health of grazing livestock [12]. Further, the acid state of metal-leak impacted area soils may increase the metal contents in plants including forages [13]. Therefore, not only does the mechanism of plants coping with iron deficiency require research, but how plants respond to excess iron also needs attention.

Transporters are involved in the absorption, transportation, redistribution, and detoxification of iron in plants. Dicotyledonous alfalfa belongs to the iron uptake strategy I, in which Fe^3+^ is reduced to the form of Fe^2+^ by Ferric reduction oxidase (FRO) [14] and absorbed by the iron-regulated transporter (IRT) [15]. Natural resistance-associated macrophage proteins (NRAMPs) are a class of metal transporters widely present in plants. NRAMP family members have been shown to be involved in iron uptake and transport in various plant species ranging from *Chlamydomonas reinhardtii* to higher plants [16,17]. NRAMPs have been proven to have the function of transporting iron in *Arabidopsis thaliana* and rice [18,19,20,21,22,23,24]. Additionally, it has been shown that NRAMPs translocate iron in *Arachis hypogaea* [25], *Solanum lycopersicum* [26], *Nicotiana tabacum* [27,28], *Medicago truncatula* [29], *Hordeum vulgare* [30], and *Prunus persica* [31]. Most NRAMP genes are preferentially expressed in vegetative tissues and reproductive organs and are induced by iron deficiency.

NRAMP proteins are subcellularly localized on the polycellular component including the membrane of plasma, tonoplast, trans-Golgi, and vesicles. In NRAMP proteins, the broad selectivity for the transition-metal ions of Mn^2+^, Fe^2+^, Co^2+^ and Ni^2+^ is emphasized [32]. The crystal structure revealed that NRAMP2 transported only Fe^2+^ in a non-electrogenic and proton-independent way [33]. The substrate gradients, protein structure, and physiological voltage determined the directionality of the substrate movement [34,35]. Many apparently quirky properties of NRAMPs may turn out to be more widespread evolutionarily [36].

As upstream genes regulate the expression of metal transporters, chelators, and related enzymes, transcription factors (TFs) also play an important role in plant iron homeostasis. The v-myb avian myeloblastosis viral oncogene homolog (MYB) TFs contain tandem repeats of a ~50 amino acid DNA-binding motif and are involved in the regulation of many aspects of plant development, hormone signaling, and metabolism. Previous studies have also shown that MYB TFs have an indispensable role in the regulation of iron homeostasis, as well as the basic helix-loop-helix (bHLH) TFs [37]. Expression of MYBs upregulated by Fe starvation suggested that MYBs may play a role in iron nutrition. The orchid R2R3-MYB gene *DwMYB2* has been shown to affect iron transport and up-regulated *AtFRO2*, *AtIRT1*, and *AtIRT2* and down-regulated *AtIREG1*, *AtFRD3* and *AtNRAMP1* [38,39]. *MYB72* and *MYB10* are all involved in the expression regulation of an important iron homeostasis-related protein, nicotinamide synthase4 [40]. Iron deficiency in the rhizosphere activates *MYB72* in the absence of shoot-derived signals. In response to volatile organic compounds, *MYB72* is co-expressed with the iron uptake-related genes *FRO2* and *IRT1* [41]. β-Glucosidase42 is an *MYB72*-dependent key regulator of rhizobacteria-induced systemic resistance and modulates iron deficiency responses in Arabidopsis roots. A total of 195 genes was constitutively upregulated in *MYB72*-overexpressing roots in the absence of *Pseudomonas fluorescens* WCS417 bacteria [42]. *MdMYB58* was shown to transcriptionally repress *MdMATE43* in apple plants and its homolog *FRD3* in Arabidopsis by directly binding to their promoter [43]. Transcriptomic analysis indicated that the control of Fe homeostasis was crucial to the response to ammonium nutrition and evidenced that *MYB28* and *MYB29* play a role in this control [44]. Overexpression of *MhR2R3-MYB4* enhanced *Arabidopsis thaliana* tolerance to Fe deficiency and led to multiple biochemical changes [45]. The *osmyb36* mutants showed higher calcium levels and lower Fe and other divalent metal-ion levels in shoots [46]. *MYB10* and *MYB7* were identified as candidates for effecting IRT1-dependent Fe mobilization in roots [47]. In humans, an HBS1L-MYB intergenic region variant associates both with an increased risk of iron overload and a reduced risk of iron deficiency anemia [48]. These results suggest the function as a regulator of iron uptake and storage of MYB TFs in plants.

Interestingly, MYB TFs can also form a regulatory network with bHLH TFs to regulate iron homeostasis. As the upstream genes regulating bHLH, Arabidopsis *MYB28*/*29* interact to negatively regulate *bHLH38/39/100/101* and *MYB10/72* [49]. *MYB10* and *MYB72* are also negatively regulated by *PYE*(*bHLH47*) [40]. *MYB72* is an intrinsic part of the plant’s iron-acquisition response in a manner that is dependent on the FER-like iron deficiency induced transcription factor (FIT). Additionally, *MdMYB58*/*43*-mediated regulation may be related to the *PYE*-related Fe deficiency regulatory network via *MdSAT1*, a member of the IVa subfamily of bHLH TFs [43]. This research highlights the fact that MYB is also an important regulator of iron homeostasis.

In this study, we cloned a *NRAMP* family gene, *MsNRAMP2*, from alfalfa. To identify the function of *MsNRAMP2,* the study on its expression, promoter activity, and heterologous expression were performed. In addition, we cloned a 1R-MYB gene, *MsMYB*, which is co-regulated with *MsNRAMP2* under iron excess. The regulatory relationship of *MsMYB* on *MsNRAMP2* was detected by a yeast one-hybrid assay. We hope the study will provide new insights into the tolerance mechanism to iron excess stress in alfalfa and iron homeostasis regulation in plants.

## 2. Results

### 2.1. Phylogenetic Analysis of NRAMP Genes

**Figure 1:**
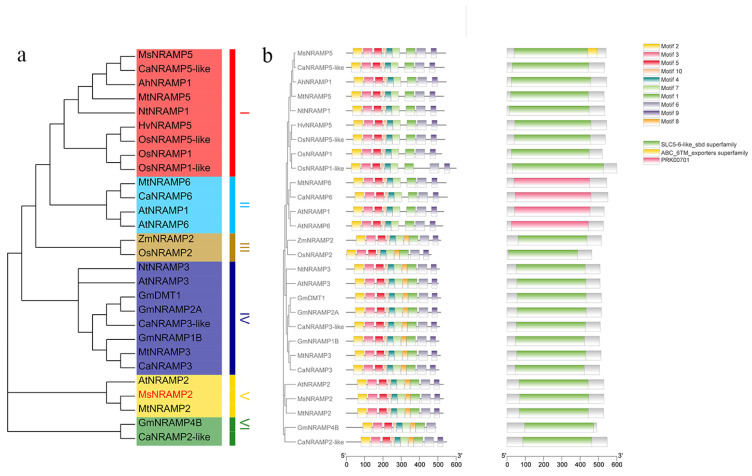
Phylogenetic Analysis of NRAMP Genes: (**a**) Phylogenetic tree constructed by *MsNRAMP2* and some NRAMP genes in *Medicago sativa* (*MsNRAMP5*: MW_762599), *Medicago truncatula* (*MtNRAMP2*: XM_013606289.3; *MtNRAMP3*: XM_003611600.4; *MtNRAMP5*: XM_003602005.4; *MtNRAMP6*: XM_003602005.4), *Arabidopsis thaliana* (*AtNRAMP1*: NM_106731.3; *AtNRAMP2*: NM_103618.3; *AtNRAMP3*: NM_127879.4; *AtNRAMP5*: NM_117995.2; *AtNRAMP6*: NM_101464.4;), *Oryza sativa* (*OsNRAMP1*: XM_015839391.1; *OsNRAMP1-like*: XM_015792140.2; *OsNRAMP2*: AAB61961.1; *OsNRAMP5-like*:XM_015789528.2), *Glycine max* (*GmNRAMP2A*: NM_001357778.1; *GmNRAMP1B*:NM_001357777.1; *GmNRAMP4B*:XM_014768591.3; *GmDMT1*: NM_001249798.2), *Nicotiana tabacum* (*NtNramp1*: XM_016629867.1; *NtNramp3-like*: XM_016614857.1), *Hordeum vulgare* (*HvNramp5*:LC184278.1), *Zea mays* (*ZmNRAMP2*: NM_001156808.2), *Arachis hypogaea* (*AhNRAMP1*: JQ_581595.1) and *Cicer arietinum* (*CaNRAMP2like*: XM_004510675.3; *CaNRAMP3*: XM_004511820.3; *CaNRAMP3-like*: XM_004509035.3; *CaNRAMP5-like*: XM_004502445.3; *CaNRAMP6*: XM_004486561.3); (**b**) The motif of *MsNRAMP* genes and promoters.

In order to analyze the phylogenetic relationship of MsNRAMP2, a phylogenetic tree was constructed (Figure 1a). The results show that MsNRAMP2, AtNRAMP2 and MtRNAMP2 have the highest homology (Figure S1), and belong to the same clade. The promoters of NRAMP family genes contain similar motifs. Except for the four genes contained in clade IV, all of the NRAMP family genes contain a SLC5-6 LIKE_SBD domain (Figure 1b).

### 2.2. Expression of MsNRAMP2 in Alfalfa Treated with Iron Excess

**Figure 2:**
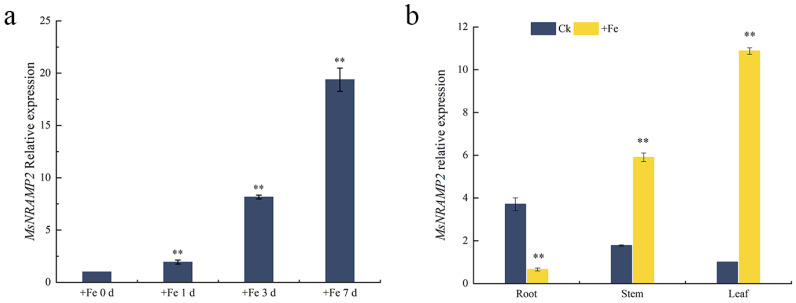
Expression pattern of *MsNRAMP2* under +Fe stress: (**a**) Relative expression of *MsNRAMP2* in whole alfalfa under different +Fe treatment durations. Alfalfa seedlings at three leaf stage were treated with Hoagland’s solution containing 460 mM iron for 0 d, 1 d, 3 d, and 7 d, respectively; (**b**) Relative expression of *MsNRAMP2* in different tissues of alfalfa under +Fe stress. Alfalfa seedlings at three leaf stage were treated with Hoagland’s solution containing 460 mM iron for 7 d, and the relative expression levels of *MsNRAMP2* in roots, stems and leaves were determined. Double asterisk indicated a significant difference from the control at *p* < 0.01 by Tukey’s test with three independent biological replicates.

The RT-qPCR was performed on *MsNRAMP2* in alfalfa plants treated with iron excess(+Fe) for 0–7 d. The results showed that the expression of *MsNRAMP2* was increased by +Fe stress in alfalfa. The expression levels of *MsNRAMP2* were significantly higher in alfalfa under +Fe treatment for 1 d, 3 d, and 7 d than that under non-treatment (Figure 2a). The results showed that *MsNRAMP2* expression was a sustained response to +Fe in 7 days. In order to analyze the expression characteristics of *MsNRAMP2*, RT-qPCR was performed in different tissues of alfalfa under +Fe treatment, with the non-treatment as the control. Compared with the control, the *MsNRAMP2* expression was significantly decreased in the roots and significantly increased in the stems and the leaves in alfalfa under +Fe treatment. These results indicate that the expression of *MsNRAMP2* was down-regulated in the roots and up-regulated in the stems and leaves by +Fe stress (Figure 2b).

### 2.3. Cloning and Characterization of MsNRAMP2

**Figure 3:**
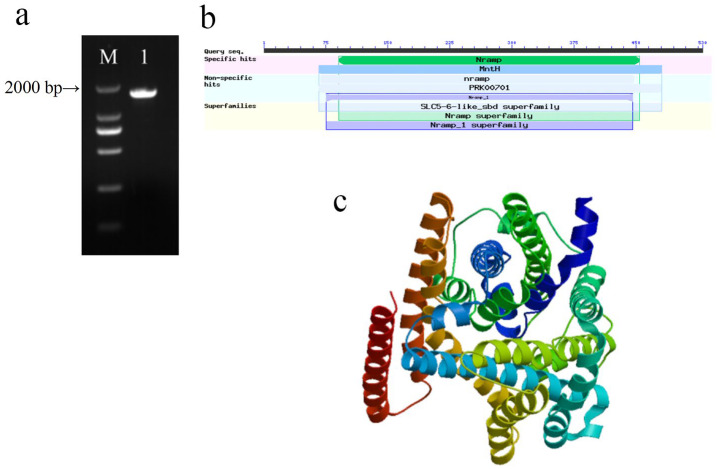
Cloning and characterization of *MsNRAMP2*: (**a**) Agarose gel electrophoresis of *MsNRAMP2* CDS PCR amplification. M: DL2000 Marker; 1: *MsNRAMP2* fragment; (**b**) Domain prediction of MsNRAMP2 protein; (**c**) Tertiary structure prediction model of MsNRAMP2 protein.

The 1593 bp CDS sequence of MsNRAMP2 (MN_547960) was cloned by PCR amplification. MsNRAMP2 protein was predicted to contain a special functional domain of Nramp (natural resistant-associated macrophage protein) and MtnH (Mn^2+^ and Fe^2+^ transporter of NRAMP family), and non-special functional domain of nramp (NRAMP metal ion transporter), PRK00701 (divalent metal cation transporter MtnH) and Nramp_1 (Nramp family divalent metal transporter). MsNRAMP2 contains SLC5-6-LIKE_SBD (solute domain of SLC5 protein), Nramp, and Nramp_1 superfamily domains (Figure 3a,b). SWISS-MODEL shows the 3D spatial model of the MsNRAMP2 protein (Figure 3c).

### 2.4. Subcellular Localization of MsNRAMP2

**Figure 4:**
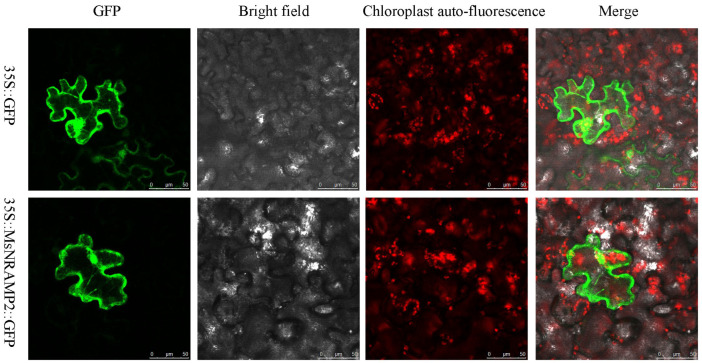
Subcellular localization of MsNRAMP2. The MsNRAMP2-GFP fusion protein was transiently expressed in the leaves of Nicotiana benthamiana (Scale bar: 50 μm).

In order to detect the subcellular localization of the MsNRAMP2 protein, a 35S::MsNRAMP2-GFP expression vector was constructed and transformed into Nicotiana benthamiana epidermal cells. The transient expression and fluorescence detection showed that the green fluorescence signals were throughout the cells in 35S::GFP transformed Nicotiana benthamiana epidermal cells, and the green fluorescence signal appeared in the nucleus and cytoplasm in 35S::MsNRAMP2-GFP transformed Nicotiana benthamiana epidermal cells. The results indicated that the MsNRAMP2 protein was scattered and distributed in the nucleus, cytoplasm, and plasma membrane (Figure 4).

### 2.5. Physiological Index in MsNRAMP2 Transformed Tobacco

**Figure 5:**
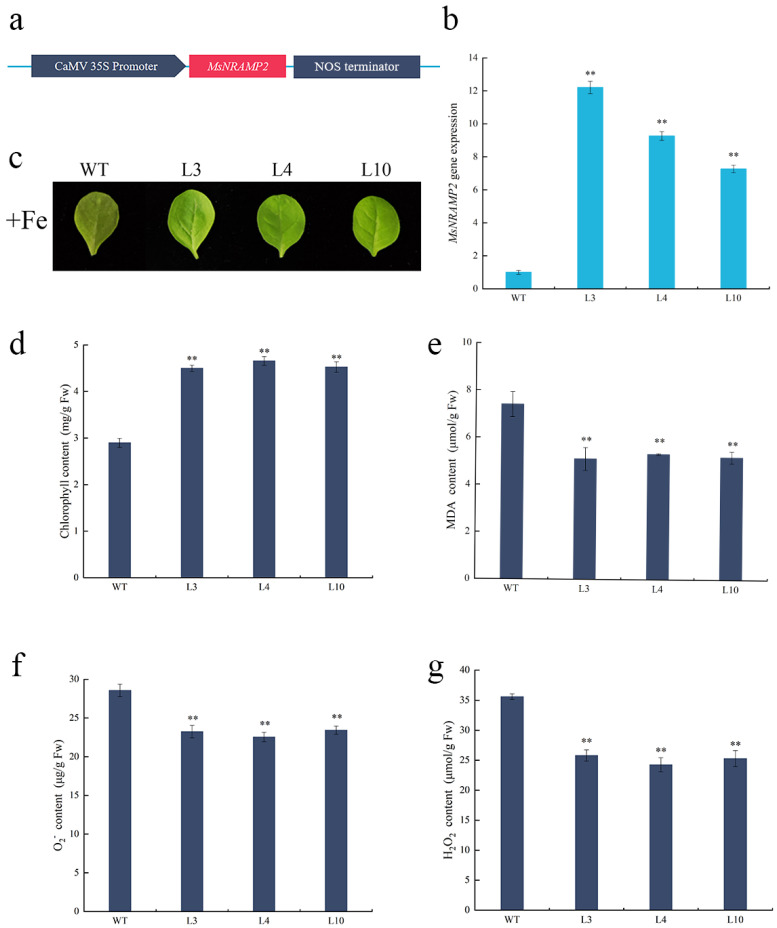
Phenotypic and physiological indices of MsNRAMP2 transgenic tobacco (L3, L4, L10) and WT under +Fe stress: (**a**) Schematic diagram of pBI121-35S::MsNRAMP2 vector construction; (**b**) Relative expression of MsNRAMP2 in transgenic tobacco; (**c**) Leaf phenotypes; (**d**) Chlorophyll content; (**e**) MDA content; (**f**) O_2_^-^ content; (**g**) H_2_O_2_ content. Double asterisk indicate significant difference from the control at *p* < 0.01 by Tukey’s test with three independent biological replicates.

In order to analyze the function of MsNRAMP2 involved in plant resistance to iron stress in tobacco, the plant expression vector pBI121-35S::MsNRAMP2 was constructed and transformed into Nicotiana tabacum by the leaf disc method mediated by Agrobacterium tumefaciens (Figure 5a). After kanamycin was screened and the PCR identified, MsNRAMP2 transgenic tobacco lines were obtained (Figure S2). The expression level of MsNRAMP2 in L3, L4 and L10 transgenic lines was significantly higher than that of WT (Figure 5b).

The MsNRAMP2 overexpression transgenic tobacco lines (L3, L4, and L10) and wild-type tobacco (WT) were treated with +Fe. After +Fe treatments for 7 d, both L3, L4, L10 and WT tobacco showed leaf yellowing. The yellowing degree is lower in L3, L4, and L10 than that in WT (Figure 5c). The chlorophyll content of L3, L4 and L10 was significantly higher than that of WT under +Fe stress (Figure 5d). These results indicated that the damage degree of chlorophyll synthesis in MsNRAMP2 overexpression tobacco was lower than that in WT under +Fe stress. The MDA, H_2_O_2_ and O_2_^-^ contents of L3, L4 and L10 were significantly lower than that of WT (Figure 5e–g). The results indicated that MsNRAMP2 overexpression tobacco sustained less membrane damage and oxidative damage under +Fe stress.

### 2.6. Growth of MsNRAMP2 Transformed Yeast

**Figure 6:**
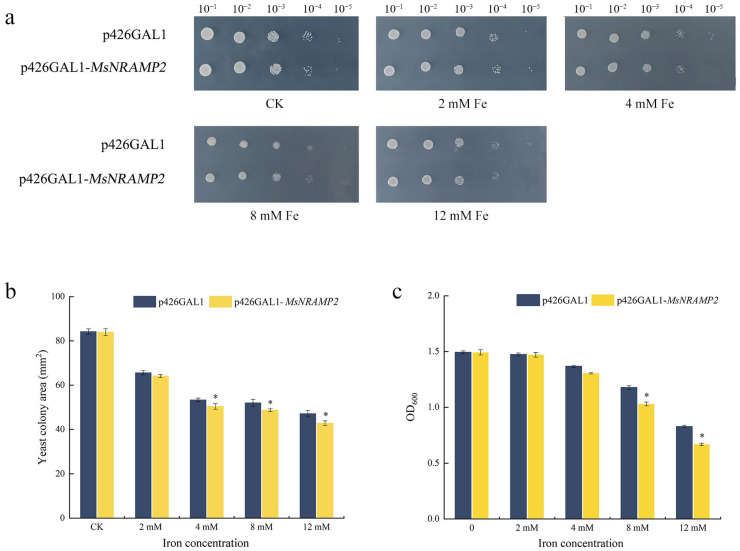
Iron tolerance function of MsNRAMP2 in yeast. The yeast containing p426GAL1 and yeast containing p426GAL1-*MsNRAMP2* were cultured on medium containing 0 mM (CK), 2 mM, 4 mM, 8 mM, and 12 mM Fe for 36 h. (**a**) Growth state of yeast; (**b**) Yeast colony area; (**c**) OD_600_ values of yeast liquid culture. Asterisk indicates significant difference from the control at *p* < 0.05 by Tukey’s test with three independent biological replicates.

The yeast expression vector p426GAL1-MsNRAMP2 was constructed and transferred into INVSC1 yeast. The p426GAL1 and P426GAL1-MsNRAMP2 transformed yeast were treated with different Fe concentrations. Under no Fe and 2 mM Fe treatments, there was no significant difference in growth between these two kinds of yeast. Under the treatment of 4 mM, 8 mM, and 12 mM Fe, no difference in growth was observed in the two kinds of yeast at 10~10^4^-fold dilutions, whereas the p426GAL1 transformed yeast grew better than P426GAL1-MsNRAMP2 transformed yeast at a 10^5^-fold dilution (Figure 6a). The sum colony area of p426GAL1 transformed yeast was significantly larger than that of p426GAL1-MsNRAMP2 transformed yeast under the treatment of 4 mM, 8 mM, and 12 mM Fe (Figure 6b). Under 2 mM, 4 mM, and no Fe treatment, there was no difference between the two kinds of yeast in OD_600_ values. When the Fe treatment concentration increased to 8 mM and 12 mM, the OD_600_ values of p426GAL1 transformed yeast liquid culture was significantly higher than that of p426GAL1-MsNRAMP2 transformed yeast liquid culture (Figure 6c). This result implies that MsNRAMP2 increases the sensitivity of yeast to iron.

### 2.7. Cloning and Characterization of MsNRAMP2 Promoter

**Figure 7:**
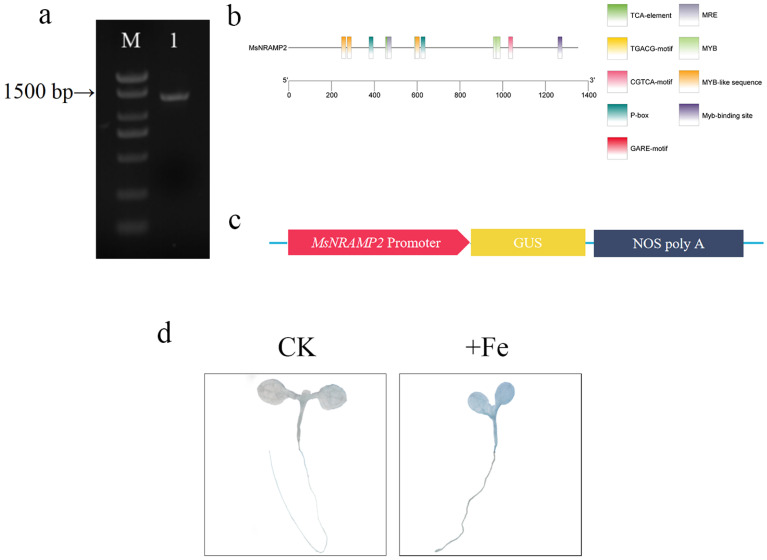
Cloning and analysis of MsNRAMP2 promoter and GUS histochemistry staining: (**a**) PCR amplification of MsNRAMP2 promoter fragment. M: DL2000 Marker; 1: MsNRAMP2 promoter fragment; (**b**) Cis-acting element prediction of the MsNRAMP2 promoter; (**c**) Schematic diagram of pBI121-pMsNRAMP2::GUS vector construction; (**d**) GUS histochemical staining in pMsNRAMP2::GUS transgenic Arabidopsis thaliana cultured under +Fe stress for 7 d, and those not treated with +Fe stress were used as control.

The 1351 bp fragment of the MsNRAMP2 promoter was cloned by PCR amplification (Figure 7a). Analysis showed that the MsNRAMP2 promoter fragment has core elements and cis-acting elements of hormone response, as well as multiple Myb-binding sites (Figure 7b). To identify the transcriptional activity of the MsNRAMP2 promoter, the pBI121-pMsNRAMP2::GUS expression vector was constructed and stable transformed into Arabidopsis thaliana (Figure 7c). The GUS staining was not obvious in the roots of Arabidopsis thaliana under +Fe treatment and non-stress conditions. There was darker blue staining in stems and leaves of Arabidopsis thaliana under +Fe treatment than those under non-stress conditions (Figure 7d). These results indicate the activity of the MsNRAMP2 promoter and suggest that the MsNRAMP2 responds to excess iron.

### 2.8. Expression Pattern Analysis and Cloning of MsMYB

**Figure 8:**
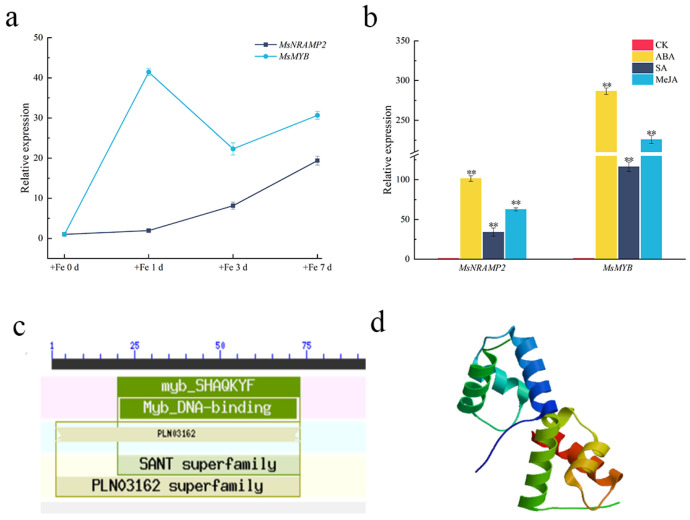
Expression pattern of MsMYB and MsNRAMP2: (**a**) The expression of MsMYB and MsNRAMP2 in alfalfa under different +Fe treatment duration. Alfalfa seedlings at three leaf stage were treated with Hoagland’s solution containing 460 mM iron for 0 d, 1 d, 3 d and 7 d, respectively; (**b**) The expression of MsMYB and MsNRAMP2 in alfalfa under ABA, SA, MeJA treatment for 1 d; (**c**) Domain prediction of MsMYB protein; (**d**) Tertiary structure prediction model of MsMYB protein. Double asterisk indicates significant difference from the control at *p* < 0.01 by Tukey’s test with three independent biological replicates.

MsNRAMP2 promoter contains many MYB binding sites and hormone response elements. We selected a MYB family transcription factor MsMYB, which is co-regulated with MsNRAMP2 in alfalfa by iron stress. The qPCR results showed that the expression of MsMYB and MsNRAMP2 was similarly induced by iron excess (Figure 8a). MsMYB and MsNRAMP2 were also similarly induced by salicylic acid, abscisic acid, and methyl jasmonate. The expression of MsMYB and MsNRAMP2 was most significantly induced by ABA (Figure 8b). The CDS of MsMYB (MN_547959) was cloned by PCR amplification (Figure S3). The obtained MsMYB gene contained 645 nucleotides, encoding 214 amino acids. The bioinformatics analysis showed that MsMYB protein contains a Myb_DNA-binding domain at the amino acids 24 to 75 and has the typical SHAQKYF domain of CCA1-like MYB, indicating that MsMYB belongs to 1R-MYB (Figure 8c). MsMYB protein had a helical, angular, and helical structure. The tertiary structure of MsMYB was consistent with the structure of MYB transcription factors (Figure 8d).

### 2.9. MsMYB Binds to MsNRAMP2 Promoter

**Figure 9:**
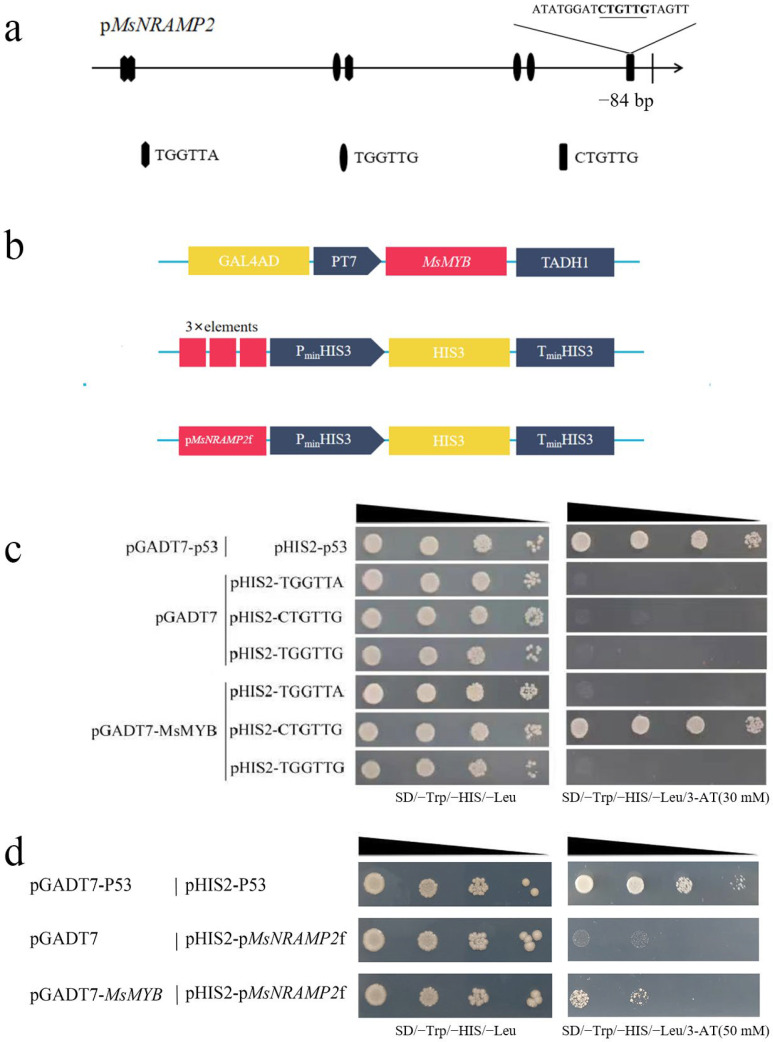
MsMYB binds to cis-elements CTGTTG and pMsNRAMP2f: (**a**) Myb-binding site in the MsNRAMP2 promoter; (**b**) Schematic diagram of pHIS2-elements, pHIS2-pMsNRAMP2f and pGADT7-Rec2-MsMYB vector construction; (**c**) Interaction analysis of three elements (TGGTTA, TGGTTG and CTGTTG) and MsMYB by Y1H; (**d**) Interaction analysis of pMsNRAMP2f and MsMYB by Y1H.

Bioinformatics analysis predicted that the MsNRAMP2 promoter fragment contained three kinds of MYB binding sites, with the sequence TGGTTA, TGGTTG, and CTGTTG (Figure 9a). In order to identify the interactions between MsMYB and MsNRAMP2, three pHIS2-element yeast expression vectors were constructed. Y1H assay was performed on MsMYB protein and three elements TGGTTA, TGGTTG, and CTGTTG in pHIS2 (Figure 9b). The results showed that MsMYB could bind to the CTGTTG element in the MsNRAMP2 promoter, while it could not bind to the other two elements (Figure 9c). To further verify, a 280 bp fragment of the MsNRAMP2 promoter containing CTGTTG element (pMsNRAMP2f) was constructed into the yeast expression vector (Figure 9b) and hybridized with MsMYB protein. The results showed that MsMYB could also bind to this 280 bp fragment of the MsNRAMP2 promoter (Figure 9d).

## 3. Discussion

Plants depend on various metal transporters and transcription factors to tolerate different iron nutrition environments. Iron(Fe) is an important essential heavy metal, while being toxic to plants when it exceeds [5].

Over recent decades, progress has been achieved in understanding the function and regulation of metal transporters in plants’ response to iron deficiency, but little is known about how plants deal with the iron excess condition. Although a large amount of research has been conducted on iron toxicity in rice [50], little research has been undertaken on iron toxicity in other crops. The effects of iron excess and the response to iron excess have been determined in soybean [51], common bean [52], radish [53], sweet potato [54,55], and wheat [56]. However, in-depth mechanism studies are still lacking. Alfalfa (*Medicago sativa* L.) is a significant forage legume cultivated worldwide and utilized in animal production. Alfalfa showed beneficial effects in iron-overload conditions and its vegetable sprouts enrich with Fe [57,58,59], which means there is a unique iron absorption and storage mechanism in alfalfa.

The natural resistance-associated macrophage protein (NRAMP) family members in plants are particularly striking for multiple divalent metal cation transporting, accepting ions including iron, aluminum, and cadmium [23,60,61,62,63]. NRAMP proteins have been reported in a number of plant species. AtNRAMP3, AtNRAMP4, and OsNRAMP1 have been shown to respond to iron deficiency stress [63,64]. The leguminous species remain largely unknown NRAMP proteins. To date, there are few studies on NRAMP gene expression induced by iron excess. In this study, *MsNRAMP2* was first isolated and characterized. The MsNRAMP2 has high homology with AtNRAMP2 in *Arabidopsis thaliana* and MtNRAMP2 in *Medicago truncatula*. AtNRAMP2 is a trans-Golgi network-localized manganese transporter that is required for Arabidopsis root growth under manganese deficiency and is critical for photosynthesis and cellular redox homeostasis [65,66]. Although manganese and iron have similar properties and often share transporters, *AtNRAMP2*’s involvement in iron deficiency or iron excess tolerance has not been reported.

The RT-qPCR and GUS assay confirmed that the expression of *MsNRAMP2* was induced by iron excess and followed the treatment time (Figure 2a and Figure 7d). Most NRAMPs are expressed in the roots and strongly induced by iron deficiency, considered to be transporters involved in iron uptake, such as AhNRAMP1, MtNRAMP1, NtNRAMP1, and NtNRAMP3 [26,28,29,30]. Interestingly, *MsNRAMP2* was upregulated in stems and leaves and downregulated in roots under iron excess (Figure 2b). The expression of *TpNRAMP3* is upregulated in leaves, while the expression of *TpNRAMP5* is downregulated in roots under iron excess stress [67,68]. The different expression patterns suggest that some potential differences probably exist in *MsNRAMP2* and its homologs, which may depend on the plant species. Thus, we hypothesize that *MsNRAMP2* may also play a role in the maintenance of iron homeostasis under iron excess in plants.

The NRAMP proteins present a broad spectrum of intracellular localizations and have a variety of potential functions. For example, AtNRAMP3/4 and GmNRAMPs are localized in the vacuolar membrane [22,69]. AtNRAMP6 is localized in the plasma membrane and intima, including the endoplasmic reticulum [70]. OsNRAMP2 and ZmNRAMP2 are tonoplast-localized transporters [71,72]. In this study, The MsNRAMP2 protein is located in the whole cell, including the nucleus and cytoplasm but not in the chloroplast (Figure 4). The results suggested that MsNRAMP2 may have a wider range of functions. Further experiments are needed to exactly determine the subcellular location of MsNRAMP2 in our subsequent studies.

In order to investigate the role of *MsNRAMP2* in response to iron excess stress, we constructed *MsNRAMP2* overexpressing tobacco lines. Usually, excessive iron in plants will cause an oxidative effect and lead to a decrease in chlorophyll content in plants [73,74]. Similar to *NtNRAMP1*, the higher chlorophyll content represented the fact that Fe excess did not damage photosynthesis in *MsNRAMP2* overexpressing tobacco lines. The lower MDA of O_2_^-^ and H_2_O_2_ content directly represent the fact that fewer reactive oxygen species accumulated in *MsNRAMP2* overexpressing tobacco lines [75]. Different from our results, the contents of MDA and H_2_O_2_ increased in tobacco overexpressed with *NtNRAMP1* [28]. The results indicated that *MsNRAMP2* enhanced tobacco tolerance to excess iron. It is suggested that *MsNRAMP2* may participate in iron redistribution and contribute to tolerance to excess iron. In order to further verify the iron tolerance function of *MsNRAMP2*, we overexpressed *MsNRAMP2* in yeast. Yeast that overexpressed *MsNRAMP2* showed greater sensitivity to iron supply (Figure 6), suggesting that *MsNRAMP2* allows more iron to enter yeast cells. The results are similar to *ZmNRAMP4*’s enhanced plant tolerance to aluminum and yeast sensitivity to aluminum [76]. In brief, *MsNRAMP2* could enhance the ability of iron tolerance and may be due to the iron transport function and/or the regulation of iron redistribution in cells.

**Figure 10:**
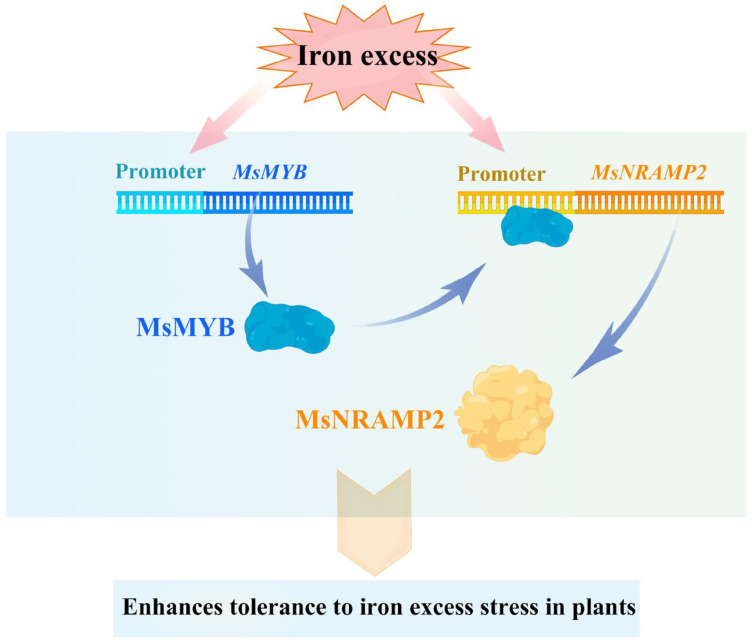
The interaction between MsNRAMP2 and MsMYB.

NRAMP is the major iron transporter in cells and is known to be regulated at the transcriptional level to intracellular iron concentrations. It revealed that the transcription factor INO plays an important role in reducing Fe loading into early-developing seeds via the repression of *NRAMP1* expression [77]. The *MsNRAMP2* promoter contains many hormone response elements and MYB binding sites (Figure 7b). We cloned a transcription factor *MsMYB* of the MYB family co-regulated with *MsNRAMP2* by iron excess (Figure 8a,b). Y1H results showed that MsMYB could bind to the *MsNRAMP2* promoter (Figure 9c,d). This result suggests that *MsNRAMP2* expression may be regulated by MsMYB. Current studies have shown that MYB transcription factors could participate in the regulation of iron homeostasis in plants by regulating the expression of transporters [39]. *MsNRAMP2* expression was significantly responsive to MeJA and SA (Figure 8b). MeJA and SA play an important role in plant resistance to abiotic stress [2]. Studies have shown that MeJA can affect the absorption of Zn, Mn, and Fe in *Arabidopsis thaliana* [78]. ABA content in plants can be induced by iron excess and ABA signal transduction pathways can also affect the expression and function of key genes in the regulatory network of iron homeostasis [79,80,81]. It is confusing that none of the known ABA-responsive element occurs in the *MsNRAMP2* promoter but *MsNRAMP2* and *MsMYB* both respond to ABA treatment (Figure 8b). This further provided indirect evidence that *MsNRAMP2* expression is regulated by MsMYB (Figure 10).

NRAMP proteins are widely involved in the resistance to iron deficiency stress, but few studies have been conducted on the resistance to iron excess stress. In this study, the function of *MsNRAMP2* under iron excess was analyzed and the regulation of its expression by MsMYB was revealed. It provides new insight into the tolerance to excess iron stress in alfalfa. At the same time, MsMYB is involved in the regulation of iron homeostasis, and the regulation function of the MYB transcription factor on iron transporters can also be the focus of future research. These findings will contribute to understanding the function of the NRAMP and MYB genes of iron homeostasis in plants.

## 4. Materials and Methods

### 4.1. Plant Materials and Growth Conditions

Alfalfa (*Medicago sativa* L. cv. Zhaodong) seeds were donated by the Animal Husbandry Institute of Heilongjiang Academy of Agricultural Sciences. The alfalfa seeds were germinated and then hydroponic cultured in 1/2 Hoagland’s solution, which was replaced every 3 d. These alfalfa were used for stress treatment and DNA and RNA extraction. Tobacco (*Nicotiana tabacum* L. cv. K326) was cultivated in soil and used for genetic transformation and physiological index detection. Nicotiana benthamiana was cultivated in soil and used for subcellular localization. Arabidopsis (*Arabidopsis thaliana* L. Col-0) was cultured in soil and used for genetic transformation and GUS histochemical staining. All plants were cultured in a 16/8 h (light/ dark, 22/20 °C) greenhouse.

### 4.2. Expression Analysis of MsNRAMP2 and MsMYB

For iron excess (+Fe) treatment, the hydroponic trefoil stage alfalfa seedlings were treated with 1/2 Hoagland’s solution containing 460 mM Fe-EDTA for 0 d, 1 d, 3 d, and 7 d. For phytohormone treatment, the hydroponic trefoil stage alfalfa seedlings were treated with 0.25 mM salicylic acid (SA), 0.1 mM abscisic acid (ABA), or 0.2 mM methyl jasmonate (MeJA) for 24 h, separately, with non-phytohormone treatment as the control. The RNA isolated from samples of whole plants, roots, stems, and leaves was used as the RT-qPCR template. The RT-qPCR was performed using SYBR^®^Premix Ex Taq^™^ (Tli RNaseH Plus) (TaKaRa Co., Ltd., Tokyo, Japan). The primers used are listed in Supplementary Table S1.

### 4.3. Cloning of MsNRAMP2, MsMYB and MsNRAMP2 Promoter

The 0.1 g of plant tissue was ground in liquid nitrogen. The total RNA was extracted using E.Z.N.A.^®^ Total RNA Kit (OMGAE Co., Ltd., Wood Dale, IL, USA) and reversed into cDNA by ReverTra Ace™ qPCR RT Master MIX (TOYOBO Co., Ltd., Osaka, Japan). The total DNA was extracted using E.Z.N.A.^®^ SP Plant DNA Kit (OMGAE Co., Ltd., Wood Dale, IL, USA).

The cloning primers are shown in Supplementary Table S1. The CDS sequences of MsNRAMP2 and MsMYB were obtained by PCR amplification using the alfalfa cDNA template under iron excess stress. The MsNRAMP2 promoter fragment was obtained by PCR amplification using alfalfa total DNA as the template. The PCR product was purified and cloned into a pMD18-T vector and sequenced (Sangon Biotech Co., Ltd., Shanghai, China).

### 4.4. Bioinformatics Analysis

The protein domain was predicted using NCBI (https://www.ncbi.nlm.nih.gov/, accessed on 20 March 2022) and the tertiary structure was predicted using SWISS MODLE (https://swissmodel.expasy.org, accessed on 28 March 2022). The phylogenetic evolutionary tree was constructed using NCBI database information, MEGA5.0 (Mega Co., Ltd., Auckland, New Zealand), and TBtools II 1.120. DNAMAN 6.0 (Lynnon Biosoft Co., Ltd., San Ramon, CA, USA) was used for the nucleotide sequence alignment. The cis-acting element in the promoter was analyzed using Plant care (https://bioinformatics.psb.ugent.be/webtools/plantcare/html, accessed on 2 May 2022).

### 4.5. Subcellular Localization of MsNRAMP2

The pBI121-MsNRAMP2-GFP recombinant vector was constructed using Xba I and Sma I by an enzyme digestion ligand. The MsNRAMP2 fragment without a stop codon was connected to the GFP and was expressed by the CaMV 35S promoter. The 35S::MsNRAMP2-GFP fusion expression vector and the control 35S::GFP vector were transformed into Agrobacterium tumefaciens GV3101. After PCR identification, the bacterial solution was injected into the 6–8 leaf stage Nicotiana benthamiana leaves. After being cultured for 72 h, the fluorescence of GFP was observed using a Leica TCS SP8 confocal microscope by laser excitation with a wavelength of 488 nm.

### 4.6. Detection of Physiological Indexes in MsNRAMP2 Overexpressing Tobacco

The pBI121-MsNRAMP2 recombinant vector was constructed. The expression of MsNRAMP2 was driven by the CaMV 35S promoter. The pBI121-MsNRAMP2 expression vector was transformed into *Agrobacterium tumefaciens* GV3101 and the leaf-disc transformation method was used to infect tobacco. The transformed tobacco seedlings were screened with 100 mg/L kanamycin, and identified by PCR and RT-qPCR. The primers were shown in Supplementary Table S1. The MsNRAMP2 transgenic tobacco was sub-generated to T_3_. Two-month-old MsNRAMP2 overexpress tobacco and wild-type tobacco were treated with 460 mM Fe-EDTA for 7 d, renewed every 2 d. The chlorophyll content was determined by the acetone method. The MDA content, H_2_O_2_ content and O_2_^-^ content were determined using the methods of spectrophotometry.

### 4.7. GUS Staining

The pCAMBIA1301-proMsNRAMP2::GUS recombinant vector was constructed and then transformed into Agrobacterium tumefaciens GV3101 to infect the plant, with the pCAMBIA1301 vector as the control. Arabidopsis was infected by the dipping method. One-week-old proMsNRAMP2::GUS Arabidopsis seedlings were cultured with 460 mM Fe-EDTA for 7d. GUS staining was performed using a GUS stain Kit (Coolaber Co., Ltd., Beijing, China).

### 4.8. Growth Detection of MsNRAMP2 in Yeast

The p426GAL1-MsNRAMP2 recombinant vector was constructed using Sac I and Kpn I by enzyme digestion ligand. The p426GAL1-MsNRAMP2 fusion expression vector and p426GAL1 empty vector were transformed into yeast (Saccharomyces cerevisiae INVSC1). The single colony was inoculated in SC-U liquid medium and incubated at 30 °C for 24 h. After culture centrifugation, 10 mL SC-U (+glucose) induction medium was used to suspend the yeast solution to OD_600_ = 0.4 density, and cultured at 30 °C for 36 h. The yeast liquid culture was collected at OD_600_ = 1.3. The yeast liquid culture was diluted 10-, 100-, 1000-, 10,000-, and 100,000-fold successively, and the yeast then diluted onto the SC-U+ glucose solid medium (0 mM, 2 mM, 4 mM, 8 mM, and 12 mM Fe). The 3 µL yeast liquid culture was used for each individual inoculation. After incubation at 30 °C for 72 h, the growth state of the colony was observed and the colony area was measured using Image J. Undiluted yeast liquid culture (OD_600_ = 1.3) was inoculated respectively into 4 mL SC-U+ glucose liquid medium containing 0 mM, 2 mM, 4 mM, 8 mM, and 12 mM Fe. After being incubated at 30 °C for 36 h, the OD_600_ value of the yeast liquid culture was determined.

### 4.9. Yeast One Hybrid

For Y1H, yeast expression vector pGADT7-Rec2-MsMYB, three kinds of pHIS2-elements (TGGTTA, TGGTTG, and CTGTTG) and pHIS2-pMsNRAMP2f were constructed by enzyme digestion and ligand method. The elements (TGGTTA, TGGTTG, and CTGTTG) were synthesized (Sangon Biotech Co., Ltd., Shanghai, China) and connected to an empty pHIS2 vector. pMsNRAMP2f was intercepted from 0 to 280 bp of the MsNRAMP2 promoter 3′ to 5′. pGADT7-Rec2-p53 and pHIS2-p53 were co-transferred into yeast Y187 as a positive control. pGADT7-Rec2 was co-transferred separately with three pHIS2-elements and pHIS2-pMsNRAMP2f into yeast Y187 as a negative control. pGADT7-Rec2-MsMYB was co-transferred separately to yeast Y187 with three pHIS2-elements and pHIS2-pMsNRAMP2f, respectively, to verify the binding of elements and the MsNRAMP2 promoter to MsMYB. After SD/-Trp/-Leu screening and PCR identification, the positive clones were diluted to the same concentration. The yeast liquid culture was diluted 10-, 100-, and 1000-fold and inoculated on the surface of the SD/-Trp/-His/-Leu and SD/-Trp/-His/-Leu/3-AT medium. After it had been cultured at 30 °C for 72 h, we observed the growth state. When the 3-AT concentration is sufficient to inhibit the growth of the negative control and the experimental group yeast can still grow, it is considered that the experimental group fragment could interact with the MsMYB protein.

### 4.10. Statistical Analysis and Reproducibility

All treatments were repeated at least three times. Statistical analysis, including a Student’s t-test, was performed using the SPSS 20 software (IBM Co., Ltd., Almonk, NY, USA).

## Data Availability

Not applicable.

## Supplementary Materials

The following supporting information can be downloaded at: https://www.mdpi.com/article/10.3390/ijms241411278/s1.

## References

[1] Radovic J., Sokolovic D., Markovic J. (2009). Alfalfa-most important perennial forage legume in animal husbandry. Biotechnol. Anim. Husb..

[2] Swiatek A., Van Dongen W., Esmans E.L., Van Onckelen H. (2004). Metabolic fate of jasmonates in tobacco bright yellow-2 cells. Plant Physiol..

[3] Chen L., Beiyuan J., Hu W., Zhang Z., Fang L. (2022). Phytoremediation of potentially toxic elements (PTEs) contaminated soils using alfalfa (*Medicago sativa* L.): A comprehensive review. Chemosphere.

[4] Khan Z., Safdar H., Ahmad K., Wajid K., Bashir H., Ugulu I., Dogan Y. (2019). Health risk assessment through determining bioaccumulation of iron in forages grown in soil irrigated with city effluent. Environ. Sci. Pollut. Res. Int..

[5] Jócsák I., Knolmajer B., Szarvas M., Rabnecz G., Pál-Fám F. (2022). Literature Review on the Effects of Heavy Metal Stress and Alleviating Possibilities through Exogenously Applied Agents in Alfalfa (*Medicago sativa* L.). Plants.

[6] Singh Dhaliwal S., Sharma V., Kumar Shukla A., Singh Shivay Y., Hossain A., Verma V., Kaur Gill M., Singh J., Singh Bhatti S., Verma G. (2023). Agronomic biofortification of forage crops with zinc and copper for enhancing nutritive potential: A systematic review. J. Sci. Food Agric..

[7] Wang M., Gong J., Bhullar N.K. (2020). Iron deficiency triggered transcriptome changes in bread wheat. Comput. Struct. Biotechnol. J..

[8] Pagani M.A., Gomez-Casati D.F. (2023). Advances in Iron Retrograde Signaling Mechanisms and Uptake Regulation in Photosynthetic Organisms. Methods Mol. Biol..

[9] Ramirez L., Simontacchi M., Murgia I., Zabaleta E., Lamattina L. (2011). Nitric oxide, nitrosyl iron complexes, ferritin and frataxin: A well equipped team to preserve plant iron homeostasis. Plant Sci..

[10] Winterbourn C.C. (1995). Toxicity of iron and hydrogen peroxide: The Fenton reaction. Toxicol. Lett..

[11] Suh H.J., Kim C.S., Lee J.Y., Jung J. (2002). Photodynamic effect of iron excess on photosystem II function in pea plants. Photochem. Photobiol..

[12] Rui-fang Z., Hong W., Ai-yong L., Xing S., Zhou D. (2013). Analysis and Evaluation of Soil Available Iron Content in Gaobeidian City of Hebei Province. J. Hebei Agric. Sci..

[13] Ge X., Khan Z.I., Chen F., Research P. (2022). A study on the contamination assessment, health risk and mobility of two heavy metals in the soil-plants-ruminants system of a typical agricultural region in the semi arid environment. Environ. Sci. Pollut. Res. Int..

[14] Ferreira D.A.P., Gaiao L.M., Kozovits A.R., Messias M.C.T.B. (2022). Evaluation of metal accumulation in the forage grass Brachiaria decumbens Stapf grown in contaminated soils with iron tailings. Integr. Environ. Assess. Manag..

[15] Van de Mortel J.E., Almar Villanueva L., Schat H., Kwekkeboom J., Coughlan S., Moerland P.D., Ver Loren van Themaat E., Koornneef M., Aarts M.G. (2006). Large Expression Differences in Genes for Iron and Zinc Homeostasis, Stress Response, and Lignin Biosynthesis Distinguish Roots of Arabidopsis thaliana and the Related Metal Hyperaccumulator *Thlaspi caerulescens*. Plant Physiol..

[16] Ishimaru Y., Suzuki M., Tsukamoto T., Suzuki K., Nakazono M., Kobayashi T., Wada Y., Watanabe S., Matsuhashi S., Takahashi M. (2010). Rice plants take up iron as an Fe^3+^-phytosiderophore and as Fe^2+^. Plant J..

[17] Rosakis A., KöSter W. (2005). Divalent metal transport in the green microalga Chlamydomonas reinhardtii is mediated by a protein similar to prokaryotic Nramp homologues. Biometals.

[18] Lampe R.H., Mann E.L., Cohen N.R., Till C.P., Thamatrakoln K., Brzezinski M.A., Bruland K.W., Twining B.S., Marchetti A. (2018). Different iron storage strategies among bloom-forming diatoms. Int. J. Biol. Macromol..

[19] Curie C., Alonso J.M., Le Jean M., Ecker J.R., Briat J.F. (2000). Involvement of NRAMP1 from Arabidopsis thaliana in iron transport. Biochem. J..

[20] Castaings L., Caquot A., Loubet S., Curie C. (2016). The high-affinity metal Transporters NRAMP1 and IRT1 Team up to Take up Iron under Sufficient Metal Provision. Sci. Rep..

[21] Tiwari M., Sharma D., Dwivedi S., Singh M., Tripathi R.D., Trivedi P.K. (2014). Expression in Arabidopsis and cellular localization reveal involvement of rice NRAMP, OsNRAMP1, in arsenic transport and tolerance. Plant Cell Environ..

[22] Lanquar V., Ramos M.S., Lelievre F., Barbier-Brygoo H., Krieger-Liszkay A., Kramer U., Thomine S. (2010). Export of vacuolar manganese by AtNRAMP3 and AtNRAMP4 is required for optimal photosynthesis and growth under manganese deficiency. Plant Physiol..

[23] Li J., Wang Y., Zheng L., Li Y., Zhang W. (2019). The Intracellular Transporter AtNRAMP6 Is Involved in Fe Homeostasis in Arabidopsis. Front. Plant Sci..

[24] Ishimaru Y., Takahashi R., Bashir K., Shimo H., Nishizawa N.K. (2012). Characterizing the role of rice NRAMP5 in Manganese, Iron and Cadmium Transport. Sci. Rep..

[25] Peris-Peris C., Serra-Cardona A., Sánchez-Sanuy F., Campo S., AriñO J., San Segundo B. (2017). Two NRAMP6 Isoforms Function as Iron and Manganese Transporters and Contribute to Disease Resistance in Rice. Mol. Plant Microbe Interact..

[26] Xiong H., Kobayashi T., Kakei Y., Senoura T., Nakazono M., Takahashi H., Nakanishi H., Shen H., Duan P., Guo X. (2012). AhNRAMP1 iron transporter is involved in iron acquisition in peanut. J. Exp. Bot..

[27] Bereczky Z., Wang H.Y., Schubert V., Ganal M., Bauer P. (2003). Differential Regulation of nramp and irt Metal Transporter Genes in Wild Type and Iron Uptake Mutants of Tomato. J. Biol. Chem..

[28] Liu W., Huo C., He L., Ji X., Yu T., Zhou Z., Song L., Yu Q., Chen J., Chen N. (2022). The NtNRAMP1 transporter is involved in cadmium and iron transport in tobacco (*Nicotiana tabacum*). Plant Physiol. Biochem..

[29] Kozak K., Papierniak-Wygladala A., Palusińska M., Barabasz A., Antosiewicz D.M. (2022). Regulation and Function of Metal Uptake Transporter NtNRAMP3 in Tobacco. Front. Plant Sci..

[30] Tejada-Jiménez M., Castro-Rodríguez R., Kryvoruchko I., Lucas M.M., Udvardi M., Imperial J., González-Guerrero M. (2015). Medicago truncatula Natural Resistance-Associated Macrophage Protein1 Is Required for Iron Uptake by Rhizobia-Infected Nodule Cells. Plant Physiol..

[31] Wang F., Zhou Z., Liu R., Gu Y., Chen S., Xu R., Chen Z.H., Shabala S. (2023). In situ mapping of ion distribution profiles and gene expression reveals interactions between hypoxia and Mn(2+)/Fe(2+) availability in barley roots. Plant Sci..

[32] Noor I., Sohail H., Zhang D., Zhu K., Shen W., Pan J., Hasanuzzaman M., Li G., Liu J. (2023). Silencing of PpNRAMP5 improves manganese toxicity tolerance in peach (*Prunus persica*) seedlings. J. Hazard. Mater..

[33] Ehrnstorfer I.A., Geertsma E.R., Pardon E., Steyaert J., Dutzler R. (2014). Crystal structure of a SLC11 (NRAMP) transporter reveals the basis for transition-metal ion transport. Nat. Struct. Mol. Biol..

[34] Buracco S., Peracino B., Cinquetti R., Signoretto E., Vollero A., Imperiali F., Castagna M., Bossi E., Bozzaro S. (2015). Dictyostelium Nramp1, which is structurally and functionally similar to mammalian DMT1 transporter, mediates phagosomal iron efflux. J. Cell Sci..

[35] Bozzi A.T., Bane L.B., Zimanyi C.M., Gaudet R. (2018). Proton co-transport and voltage dependence enforce unidirectional metal transport in an Nramp transporter. BioRxiv.

[36] Bozzi A.T., McCabe A.L., Barnett B.C., Gaudet R. (2020). Transmembrane helix 6b links proton and metal release pathways and drives conformational change in an Nramp-family transition metal transporter. J. Biol. Chem..

[37] Bozzi A.T., Gaudet R. (2021). Molecular Mechanism of Nramp-Family Transition Metal Transport. J. Mol. Biol..

[38] Gao F., Robe K., Gaymard F., Izquierdo E., Dubos C. (2019). The Transcriptional Control of Iron Homeostasis in Plants: A Tale of bHLH Transcription Factors?. Front. Plant Sci..

[39] Chen Y.H., Wu X.M., Ling H.Q., Yang W.C. (2006). Transgenic expression of DwMYB2 impairs iron transport from root to shoot in Arabidopsis thaliana. Cell Res..

[40] Shen J., Xu X., Li T., Cao D., Han Z. (2008). An MYB Transcription Factor from Malus xiaojinensis Has a Potential Role in Iron Nutrition. J. Integr. Plant Biol..

[41] Long T.A., Tsukagoshi H., Busch W., Lahner B., Salt D.E., Benfey P.N. (2010). The bHLH Transcription Factor POPEYE Regulates Response to Iron Deficiency in Arabidopsis Roots. Plant Cell.

[42] Zamioudis C., Korteland J., Van Pelt J.A., van Hamersveld M., Dombrowski N., Bai Y., Hanson J., Van Verk M.C., Ling H.Q., Schulze-Lefert P. (2015). Rhizobacterial volatiles and photosynthesis-related signals coordinate MYB72 expression in Arabidopsis roots during onset of induced systemic resistance and iron-deficiency responses. Plant J..

[43] Zamioudis C., Hanson J., Pieterse C.M. (2014). β-Glucosidase BGLU42 is a MYB72-dependent key regulator of rhizobacteria-induced systemic resistance and modulates iron deficiency responses in Arabidopsis roots. New Phytol..

[44] Wang F.P., Wang X.F., Zhang J., Ma F., Hao Y.J. (2018). MdMYB58 Modulates Fe Homeostasis by Directly Binding to the MdMATE43 Promoter in Plants. Plant Cell Physiol..

[45] Coleto I., Bejarano I., Marín-Peña A.J., Medina J., Rioja C., Burow M., Marino D. (2021). Arabidopsis thaliana transcription factors MYB28 and MYB29 shape ammonium stress responses by regulating Fe homeostasis. New Phytol..

[46] Zhang Z.X., Zhang R., Wang S.C., Zhang D., Zhao T., Liu B., Wang Y.X., Wu Y.X. (2022). Identification of Malus halliana R2R3-MYB gene family under iron deficiency stress and functional characteristics of MhR2R3-MYB4 in Arabidopsis thaliana. Plant Biol..

[47] Wang Z., Zhang B., Chen Z., Wu M., Chao D., Wei Q., Xin Y., Li L., Ming Z., Xia J. (2022). Three OsMYB36 members redundantly regulate Casparian strip formation at the root endodermis. Plant Cell.

[48] Quintana J., Bernal M., Scholle M., Holländer-Czytko H., Nguyen N.T., Piotrowski M., Mendoza-Cózatl D.G., Haydon M.J., Krämer U. (2022). Root-to-shoot iron partitioning in Arabidopsis requires IRON-REGULATED TRANSPORTER1 (IRT1) protein but not its iron(II) transport function. Plant J..

[49] Bell S., Rigas A.S., Magnusson M.K., Ferkingstad E., Allara E., Bjornsdottir G., Ramond A., Sørensen E., Halldorsson G.H., Paul D.S. (2021). A genome-wide meta-analysis yields 46 new loci associating with biomarkers of iron homeostasis. Commun. Biol..

[50] Aung M.S., Masuda H. (2020). How Does Rice Defend Against Excess Iron?: Physiological and Molecular Mechanisms. Front. Plant Sci..

[51] Lapaz A.M., de Camargos L.S., Yoshida C.H.P., Firmino A.C., de Figueiredo P.A.M., Aguilar J.V., Nicolai A.B., Silva de Paiva W.D., Cruz V.H., Tomaz R.S. (2020). Response of soybean to soil waterlogging associated with iron excess in the reproductive stage. Physiol. Mol. Biol. Plants.

[52] DeLaat D.M., Colombo C.A., Chiorato A.F., Carbonell S.A. (2014). Induction of ferritin synthesis by water deficit and iron excess in common bean (*Phaseolus vulgaris* L.). Mol. Biol. Rep..

[53] Smolik B., Cichocka J., Materny A., Śnioszek M., Zakrzewska H. (2013). Effect of iron deficiency and excess on biometric and biochemical parameters indicated in the radish sprouts (*Raphanus sativus* L. Subvar. radicula pers.)/Wpyw niedoboru i nadmiaru elaza na parametry biometryczne i biochemiczne oznaczone w kiekach rzodkie. Ochr. Srodowiska Zasobów Nat..

[54] Adamski J.M., Peters J.A., Danieloski R., Bacarin M.A. (2011). Excess iron-induced changes in the photosynthetic characteristics of sweet potato. J. Plant Physiol..

[55] Adamski J.M., Danieloski R., Deuner S., Braga E.J.B., Castro L.A.S.D., Peters J.A. (2012). Responses to excess iron in sweet potato: Impacts on growth, enzyme activities, mineral concentrations, and anatomy. Acta Physiol. Plant.

[56] Li X., Ma H., Jia P., Wang J., Jia L., Zhang T., Yang Y., Chen H., Wei X. (2012). Responses of seedling growth and antioxidant activity to excess iron and copper in *Triticum aestivum* L. Ecotoxicol. Environ. Saf..

[57] Patel R., Tirgar P. (2013). Evaluation of Beneficial Effects of *Medicago Sativa* (Alfalfa) In Iron-Overload Conditions. J. Chem. Biol. Phys. Sci..

[58] Zhang H., Cao Y., Tian Y., Zheng L., Huang H. (2021). Metal speciation distribution of anaerobic fermentation with alfalfa grass harvested from abandoned iron mine and the influence of metals addition. Process. Saf. Environ. Prot..

[59] Przybysz A., Wrochna M., MalEcka-Przybysz M., Gawrońska H., Gawroński S. (2016). Vegetable sprouts enriched with iron: Effects on yield, ROS generation and antioxidative system. Sci. Hortic..

[60] Nevo Y., Nelson N. (2006). The NRAMP family of metal-ion transporters. Biochim. Biophys. Acta Bioenerg..

[61] Cailliatte R., Lapeyre B., Briat J.F., Mari S., Curie C. (2009). The NRAMP6 metal transporter contributes to cadmium toxicity. Biochem. J..

[62] Yamaji N., Sasaki A., Xia J.X., Yokosho K., Ma J.F. (2013). A node-based switch for preferential distribution of manganese in rice. Nat. Commun..

[63] Mary V., Schnell Ramos M., Gillet C., Socha A.L., Giraudat J., Agorio A., Merlot S., Clairet C., Kim S.A., Punshon T. (2015). Bypassing Iron Storage in Endodermal Vacuoles Rescues the Iron Mobilization Defect in the natural resistance associated-macrophage protein3natural resistance associated-macrophage protein4 Double Mutant. Plant Physiol..

[64] Chang J.D., Huang S., Yamaji N., Zhang W., Ma J.F., Zhao F.J. (2020). OsNRAMP1 transporter contributes to cadmium and manganese uptake in rice. Plant Cell Environ..

[65] Alejandro S., Cailliatte R., Alcon C., Dirick L., Domergue F., Correia D., Castaings L., Briat J.F., Mari S., Curie C. (2017). Intracellular Distribution of Manganese by the Trans-Golgi Network Transporter NRAMP2 Is Critical for Photosynthesis and Cellular Redox Homeostasis. Plant Cell.

[66] Gao H., Xie W., Yang C., Xu J., Li J., Wang H., Chen X., Huang C.F. (2018). NRAMP2, a trans-Golgi network-localized manganese transporter, is required for Arabidopsis root growth under manganese deficiency. New Phytol..

[67] Peng F., Wang C., Zhu J., Zeng J., Kang H., Fan X., Sha L., Zhang H., Zhou Y., Wang Y. (2018). Expression of TpNRAMP5, a metal transporter from Polish wheat (*Triticum polonicum* L.), enhances the accumulation of Cd, Co and Mn in transgenic Arabidopsis plants. Planta.

[68] Peng F., Wang C., Cheng Y., Kang H., Fan X., Sha L., Zhang H., Zeng J., Zhou Y., Wang Y. (2018). Cloning and Characterization of TpNRAMP3, a Metal Transporter From Polish Wheat (*Triticum polonicum* L.). Front. Plant Sci..

[69] Qin L., Han P., Chen L., Walk T.C., Li Y., Hu X., Xie L., Liao H., Liao X. (2017). Genome-Wide Identification and Expression Analysis of NRAMP Family Genes in Soybean (*Glycine Max* L.). Front. Plant Sci..

[70] Li L., Zhu Z., Liao Y., Yang C., Fan N., Zhang J., Yamaji N., Dirick L., Ma J.F., Curie C. (2022). NRAMP6 and NRAMP1 cooperatively regulate root growth and manganese translocation under manganese deficiency in Arabidopsis. Plant J..

[71] Li Y., Li J., Yu Y., Dai X., Gong C., Gu D., Xu E., Liu Y., Zou Y., Zhang P. (2021). The tonoplast-localized transporter OsNRAMP2 is involved in iron homeostasis and affects seed germination in rice. J. Exp. Bot..

[72] Guo J., Long L., Chen A., Dong X., Liu Z., Chen L., Wang J., Yuan L. (2022). Tonoplast-localized transporter ZmNRAMP2 confers root-to-shoot translocation of manganese in maize. Plant Physiol..

[73] Kirk G.J.D., Manwaring H.R., Ueda Y., Semwal V.K., Wissuwa M. (2022). Below-ground plant-soil interactions affecting adaptations of rice to iron toxicity. Plant Cell Environ..

[74] Guerinot M.L., Yi Y. (1994). Iron: Nutritious, Noxious, and Not Readily Available. Plant Physiol..

[75] Ranieri A., Castagna A., Baldan B., Soldatini G.F. (2001). Iron deficiency differently affects peroxidase isoforms in sunflower. J. Exp. Bot..

[76] Li H., Wang N., Hu W., Yan W., Jin X., Yu Y., Du C., Liu C., He W., Zhang S. (2022). ZmNRAMP4 Enhances the Tolerance to Aluminum Stress in Arabidopsis thaliana. Int. J. Mol. Sci..

[77] Sun L., Wei Y.Q., Wu K.H., Yan J.Y., Xu J.N., Wu Y.R., Li G.X., Xu J.M., Harberd N.P., Ding Z.J. (2021). Restriction of iron loading into developing seeds by a YABBY transcription factor safeguards successful reproduction in Arabidopsis. Mol. Plant.

[78] Li C., Wang P., Menzies N.W., Lombi E., Kopittke P.M. (2017). Effects of changes in leaf properties mediated by methyl jasmonate (MeJA) on foliar absorption of Zn, Mn and Fe. Ann. Bot..

[79] Yamaguchi-Shinozaki K., Shinozaki K. (2006). Transcriptional regulatory networks in cellular responses and tolerance to dehydration and cold stresses. Annu. Rev. Plant Biol..

[80] Munemasa S., Hauser F., Park J., Waadt R., Brandt B., Schroeder J.I. (2015). Mechanisms of abscisic acid-mediated control of stomatal aperture. Curr. Opin. Plant Biol..

[81] Zhu J.K. (2016). Abiotic Stress Signaling and Responses in Plants. Cell.

