# Supplementary material for: MsNRAMP2 Enhances Tolerance to Iron Excess Stress in Nicotiana tabacum and MsMYB Binds to Its Promoter
Source: Int J Mol Sci. 2023 Jul 10;24(14):11278. doi: 10.3390/ijms241411278 (PMC10379929; doi:10.3390/ijms241411278)
Supplement: Supplementary file 1 [file ijms-24-11278-s001.zip › Supplementary fig.pdf]

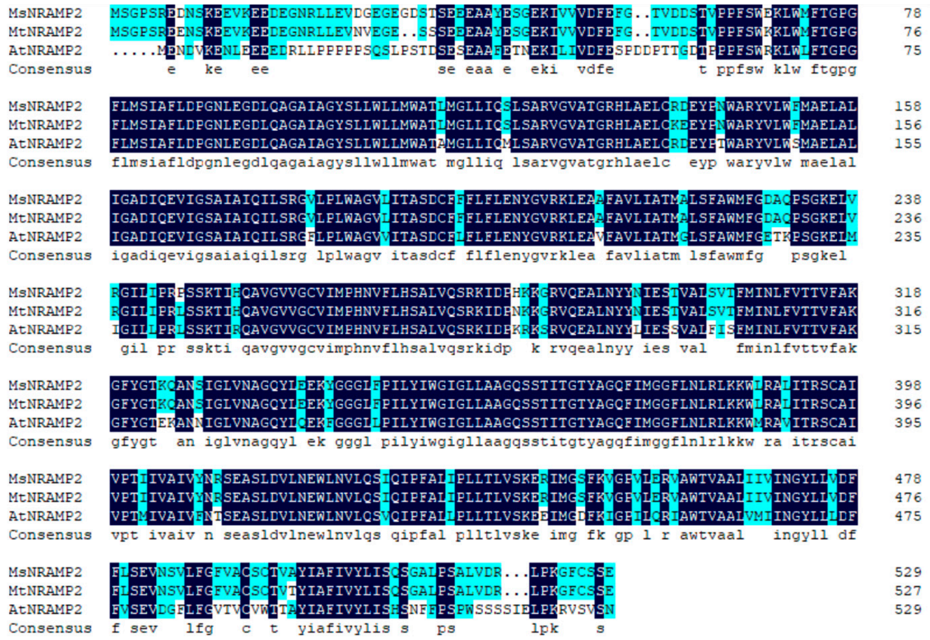

Figure S1. *MsNRAMP2* , *MtNRAMP2*(XM_013606289.3) and *AtNRAMP2*(NM_103618.3) amino acid sequence alignment.

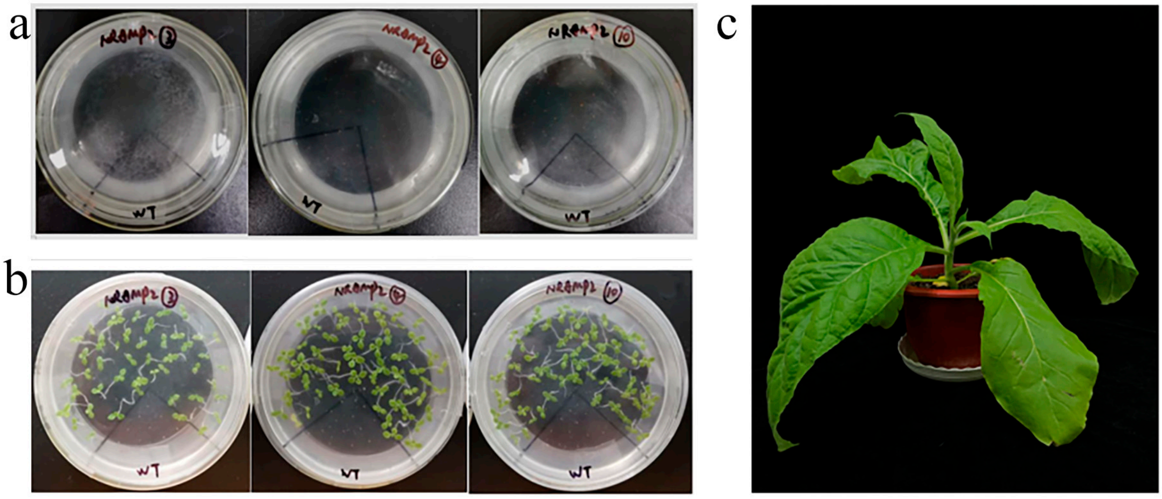

Figure S2. *MsNRAMP2* transgenic tobacco: (**a**) Germination of *MsNRAMP2* overexpression transgenic tobacco; (**b**) Screening and culture of *MsNRAMP2* transgenic tobacco; (**c**) *MsNRAMP2* transgenic tobacco positive plant.

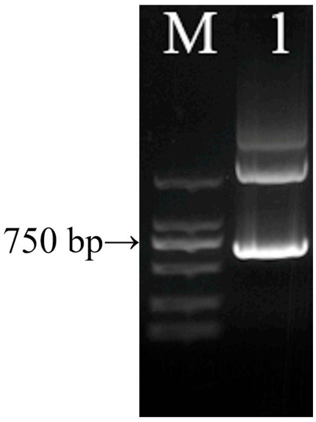

Figure S3. PCR amplification of *MsMYB* CDS fragment: M: DL2000 Marker; 1: *MsMYB* fragment.
